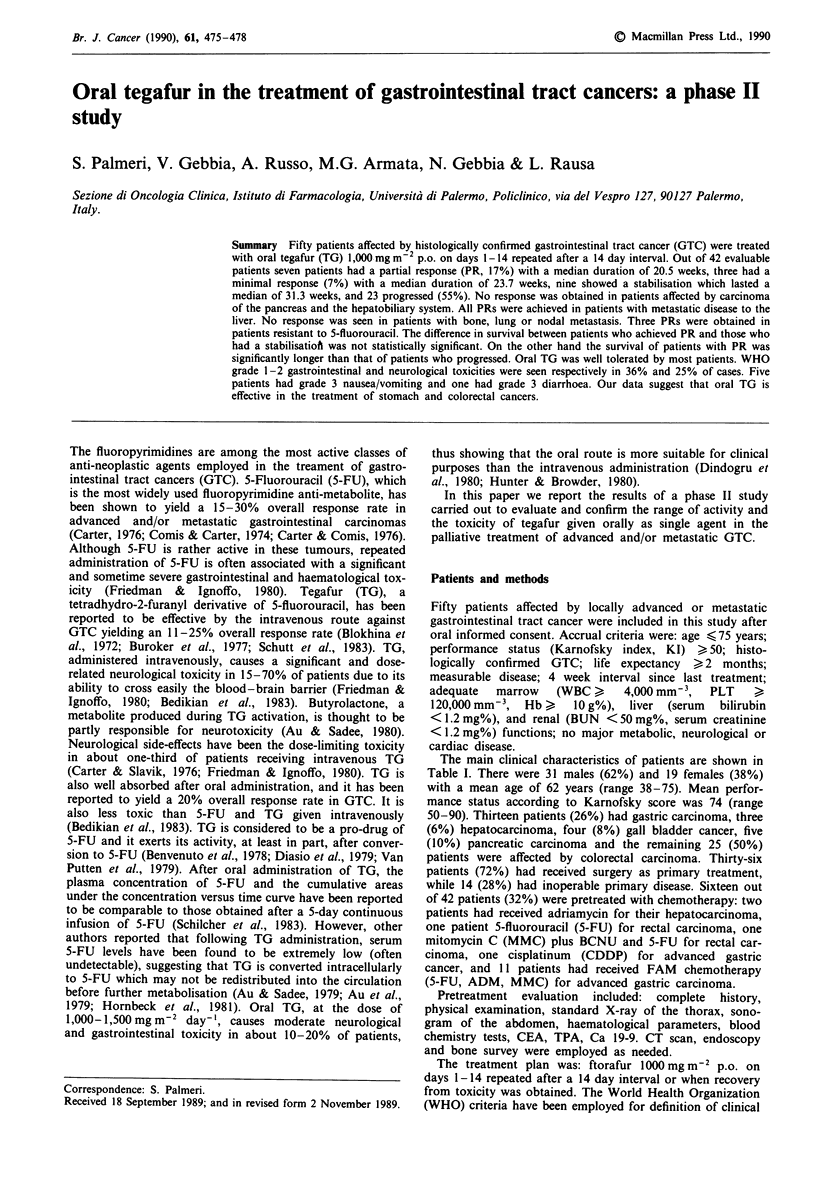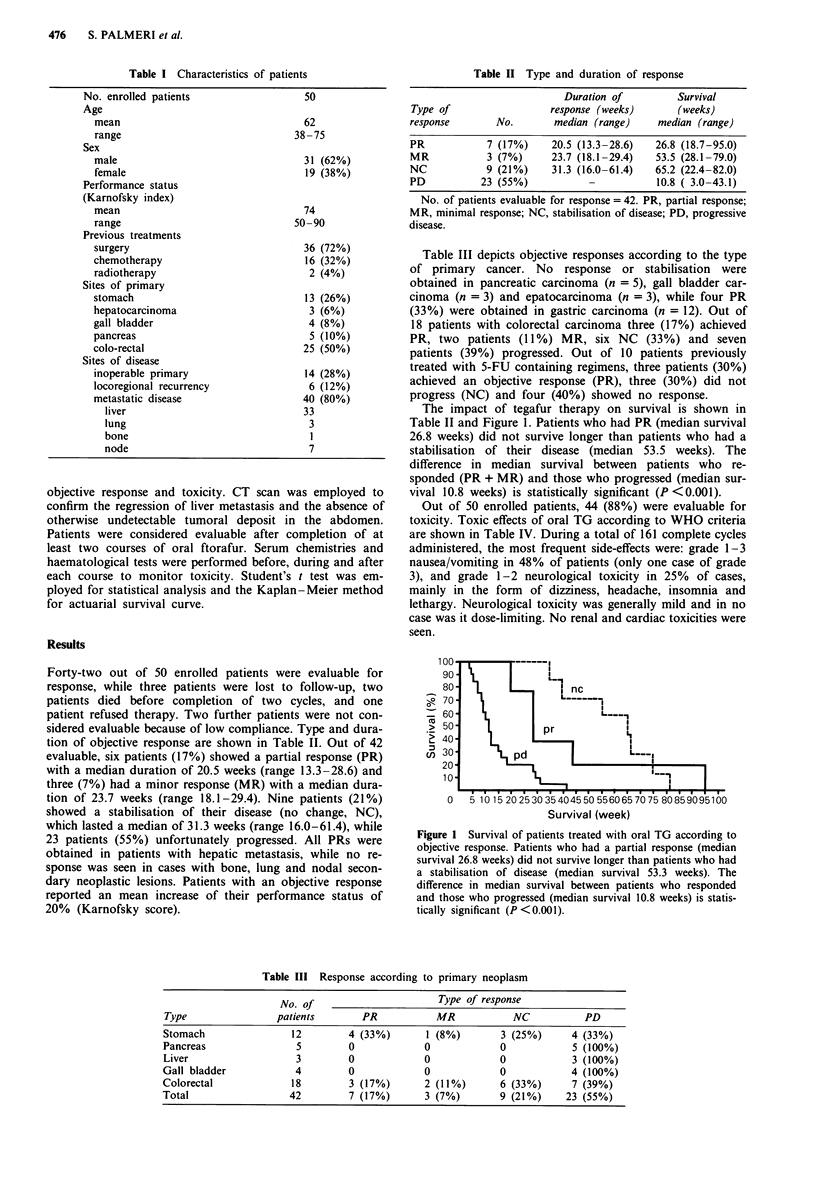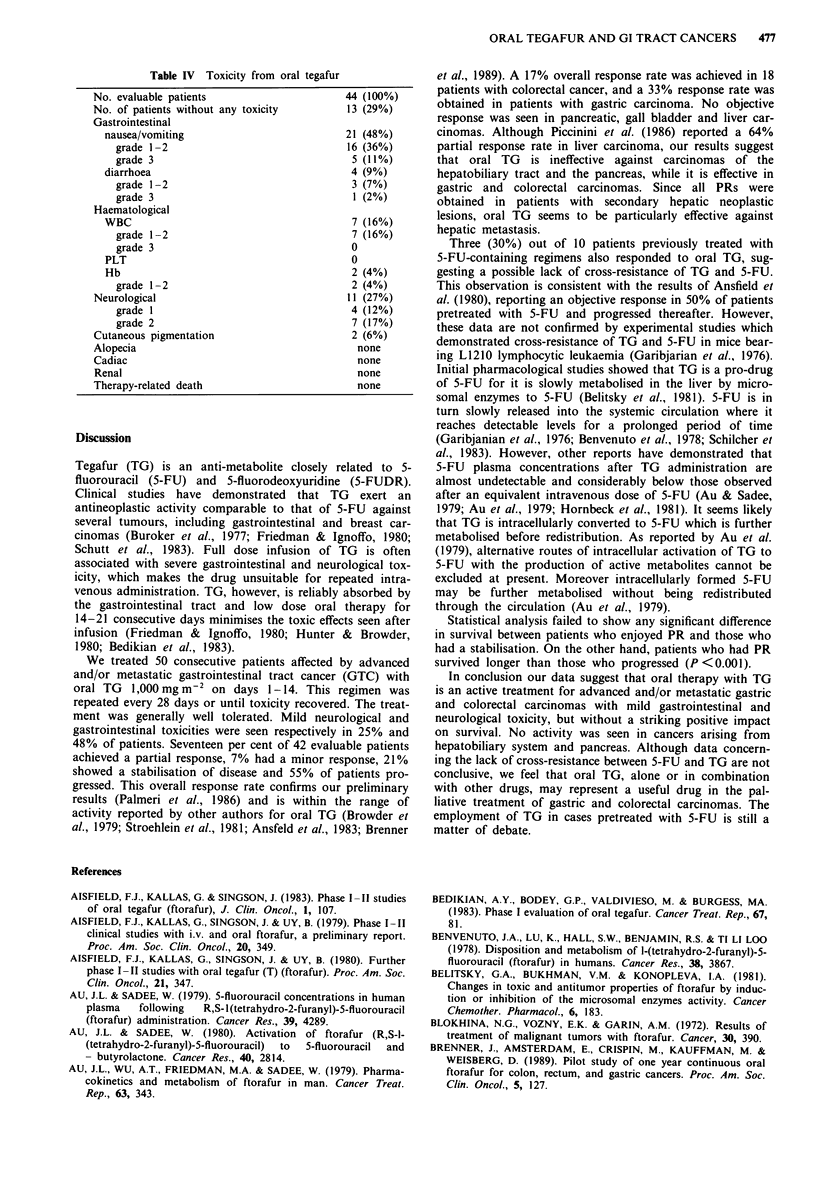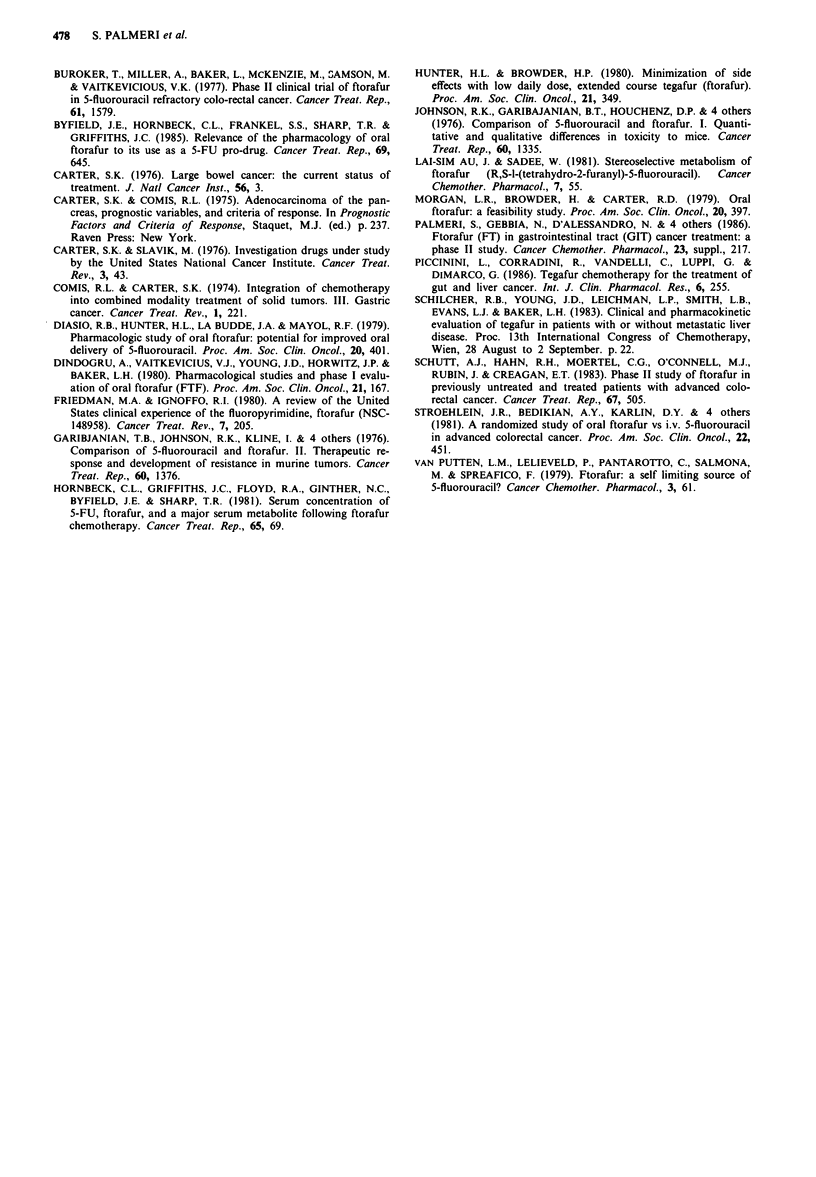# Oral tegafur in the treatment of gastrointestinal tract cancers: a phase II study.

**DOI:** 10.1038/bjc.1990.105

**Published:** 1990-03

**Authors:** S. Palmeri, V. Gebbia, A. Russo, M. G. Armata, N. Gebbia, L. Rausa

**Affiliations:** Sezione di Oncologia Clinica, Università di Palermo, Italy.

## Abstract

Fifty patients affected by histologically confirmed gastrointestinal tract cancer (GTC) were treated with oral tegafur (TG) 1,000 mg m-2 p.o. on days 1-14 repeated after a 14 day interval. Out of 42 evaluable patients seven patients had a partial response (PR. 17%) with a median duration of 20.5 weeks, three had a minimal response (7%) with a median duration of 23.7 weeks, nine showed a stabilisation which lasted a median of 31.3 weeks, and 23 progressed (55%). No response was obtained in patients affected by carcinoma of the pancreas and the hepatobiliary system. All PRs were achieved in patients with metastatic disease to the liver. No response was seen in patients with bone, lung or nodal metastasis. Three PRs were obtained in patients resistant to 5-fluorouracil. The difference in survival between patients who achieved PR and those who had a stabilisation was not statistically significant. On the other hand the survival of patients with PR was significantly longer than that of patients who progressed. Oral TG was well tolerated by most patients. WHO grade 1-2 gastrointestinal and neurological toxicities were seen respectively in 36% and 25% of cases. Five patients had grade 3 nausea/vomiting and one had grade 3 diarrhoea. Our data suggest that oral TG is effective in the treatment of stomach and colorectal cancers.


Br. J. Cancer (1990), 61, 475–478 © Macmillan Press Ltd., 1990

### Oral tegafur in the treatment of gastrointestinal tract cancers: a phase II study

S. Palmeri, V. Gebbia, A. Russo, M.G. Armata, N. Gebbia & L. Rausa

*Sezione di Oncologia Clinica, Istituto di Farmacologia, Università di Palermo, Policlinico, via del Vespro 127, 90127 Palermo, Italy.*

**Summary** Fifty patients affected by histologically confirmed gastrointestinal tract cancer (GTC) were treated with oral tegafur (TG) 1,000 mg $m^{-2}$ p.o. on days 1–14 repeated after a 14 day interval. Out of 42 evaluable patients seven patients had a partial response (PR, 17%) with a median duration of 20.5 weeks, three had a minimal response (7%) with a median duration of 23.7 weeks, nine showed a stabilisation which lasted a median of 31.3 weeks, and 23 progressed (55%). No response was obtained in patients affected by carcinoma of the pancreas and the hepatobiliary system. All PRs were achieved in patients with metastatic disease to the liver. No response was seen in patients with bone, lung or nodal metastasis. Three PRs were obtained in patients resistant to 5-fluorouracil. The difference in survival between patients who achieved PR and those who had a stabilisation was not statistically significant. On the other hand the survival of patients with PR was significantly longer than that of patients who progressed. Oral TG was well tolerated by most patients. WHO grade 1–2 gastrointestinal and neurological toxicities were seen respectively in 36% and 25% of cases. Five patients had grade 3 nausea/vomiting and one had grade 3 diarrhoea. Our data suggest that oral TG is effective in the treatment of stomach and colorectal cancers.

The fluoropyrimidines are among the most active classes of anti-neoplastic agents employed in the treament of gastro-intestinal tract cancers (GTC). 5-Fluorouracil (5-FU), which is the most widely used fluoropyrimidine anti-metabolite, has been shown to yield a 15–30% overall response rate in advanced and/or metastatic gastrointestinal carcinomas (Carter, 1976; Comis & Carter, 1974; Carter & Comis, 1976). Although 5-FU is rather active in these tumours, repeated administration of 5-FU is often associated with a significant and sometime severe gastrointestinal and haematological toxicity (Friedman & Ignoffo, 1980). Tegafur (TG), a tetradhydro-2-furanyl derivative of 5-fluorouracil, has been reported to be effective by the intravenous route against GTC yielding an 11–25% overall response rate (Blokhina *et al.*, 1972; Buroker *et al.*, 1977; Schutt *et al.*, 1983). TG, administered intravenously, causes a significant and dose-related neurological toxicity in 15–70% of patients due to its ability to cross easily the blood–brain barrier (Friedman & Ignoffo, 1980; Bedikian *et al.*, 1983). Butyrolactone, a metabolite produced during TG activation, is thought to be partly responsible for neurotoxicity (Au & Sadee, 1980). Neurological side-effects have been the dose-limiting toxicity in about one-third of patients receiving intravenous TG (Carter & Slavik, 1976; Friedman & Ignoffo, 1980). TG is also well absorbed after oral administration, and it has been reported to yield a 20% overall response rate in GTC. It is also less toxic than 5-FU and TG given intravenously (Bedikian *et al.*, 1983). TG is considered to be a pro-drug of 5-FU and it exerts its activity, at least in part, after conversion to 5-FU (Benvenuto *et al.*, 1978; Diasio *et al.*, 1979; Van Putten *et al.*, 1979). After oral administration of TG, the plasma concentration of 5-FU and the cumulative areas under the concentration versus time curve have been reported to be comparable to those obtained after a 5-day continuous infusion of 5-FU (Schilcher *et al.*, 1983). However, other authors reported that following TG administration, serum 5-FU levels have been found to be extremely low (often undetectable), suggesting that TG is converted intracellularly to 5-FU which may not be redistributed into the circulation before further metabolisation (Au & Sadee, 1979; Au *et al.*, 1979; Hornbeck *et al.*, 1981). Oral TG, at the dose of 1,000–1,500 mg $m^{-2}$ $day^{-1}$, causes moderate neurological and gastrointestinal toxicity in about 10–20% of patients, thus showing that the oral route is more suitable for clinical purposes than the intravenous administration (Dindogru *et al.*, 1980; Hunter & Browder, 1980).

In this paper we report the results of a phase II study carried out to evaluate and confirm the range of activity and the toxicity of tegafur given orally as single agent in the palliative treatment of advanced and/or metastatic GTC.

### Patients and methods

Fifty patients affected by locally advanced or metastatic gastrointestinal tract cancer were included in this study after oral informed consent. Accrual criteria were: age $\leqslant$75 years; performance status (Karnofsky index, KI) $\geqslant$50; histologically confirmed GTC; life expectancy $\geqslant$2 months; measurable disease; 4 week interval since last treatment; adequate marrow (WBC $\geqslant$ 4,000 $mm^{-3}$, PLT $\geqslant$ 120,000 $mm^{-3}$, Hb $\geqslant$ 10 g%), liver (serum bilirubin $<$1.2 mg%), and renal (BUN $<$50 mg%, serum creatinine $<$1.2 mg%) functions; no major metabolic, neurological or cardiac disease.

The main clinical characteristics of patients are shown in Table I. There were 31 males (62%) and 19 females (38%) with a mean age of 62 years (range 38–75). Mean performance status according to Karnofsky score was 74 (range 50–90). Thirteen patients (26%) had gastric carcinoma, three (6%) hepatocarcinoma, four (8%) gall bladder cancer, five (10%) pancreatic carcinoma and the remaining 25 (50%) patients were affected by colorectal carcinoma. Thirty-six patients (72%) had received surgery as primary treatment, while 14 (28%) had inoperable primary disease. Sixteen out of 42 patients (32%) were pretreated with chemotherapy: two patients had received adriamycin for their hepatocarcinoma, one patient 5-fluorouracil (5-FU) for rectal carcinoma, one mitomycin C (MMC) plus BCNU and 5-FU for rectal carcinoma, one cisplatinum (CDDP) for advanced gastric cancer, and 11 patients had received FAM chemotherapy (5-FU, ADM, MMC) for advanced gastric carcinoma.

Pretreatment evaluation included: complete history, physical examination, standard X-ray of the thorax, sonogram of the abdomen, haematological parameters, blood chemistry tests, CEA, TPA, Ca 19-9. CT scan, endoscopy and bone survey were employed as needed.

The treatment plan was: ftorafur 1000 mg $m^{-2}$ p.o. on days 1–14 repeated after a 14 day interval or when recovery from toxicity was obtained. The World Health Organization (WHO) criteria have been employed for definition of clinical

Correspondence: S. Palmeri.
Received 18 September 1989; and in revised form 2 November 1989.

**Table I** Characteristics of patients

| | |
|---|---|
| No. enrolled patients | 50 |
| Age | |
| mean | 62 |
| range | 38-75 |
| Sex | |
| male | 31 (62%) |
| female | 19 (38%) |
| Performance status (Karnofsky index) | |
| mean | 74 |
| range | 50-90 |
| Previous treatments | |
| surgery | 36 (72%) |
| chemotherapy | 16 (32%) |
| radiotherapy | 2 (4%) |
| Sites of primary | |
| stomach | 13 (26%) |
| hepatocarcinoma | 3 (6%) |
| gall bladder | 4 (8%) |
| pancreas | 5 (10%) |
| colo-rectal | 25 (50%) |
| Sites of disease | |
| inoperable primary | 14 (28%) |
| locoregional recurrency | 6 (12%) |
| metastatic disease | 40 (80%) |
| liver | 33 |
| lung | 3 |
| bone | 1 |
| node | 7 |

objective response and toxicity. CT scan was employed to confirm the regression of liver metastasis and the absence of otherwise undetectable tumoral deposit in the abdomen. Patients were considered evaluable after completion of at least two courses of oral ftorafur. Serum chemistries and haematological tests were performed before, during and after each course to monitor toxicity. Student's *t* test was employed for statistical analysis and the Kaplan-Meier method for actuarial survival curve.

### Results

Forty-two out of 50 enrolled patients were evaluable for response, while three patients were lost to follow-up, two patients died before completion of two cycles, and one patient refused therapy. Two further patients were not considered evaluable because of low compliance. Type and duration of objective response are shown in Table II. Out of 42 evaluable, six patients (17%) showed a partial response (PR) with a median duration of 20.5 weeks (range 13.3-28.6) and three (7%) had a minor response (MR) with a median duration of 23.7 weeks (range 18.1-29.4). Nine patients (21%) showed a stabilisation of their disease (no change, NC), which lasted a median of 31.3 weeks (range 16.0-61.4), while 23 patients (55%) unfortunately progressed. All PRs were obtained in patients with hepatic metastasis, while no response was seen in cases with bone, lung and nodal secondary neoplastic lesions. Patients with an objective response reported an mean increase of their performance status of 20% (Karnofsky score).

**Table II** Type and duration of response

| *Type of response* | *No.* | *Duration of response (weeks) median (range)* | *Survival (weeks) median (range)* |
|---|---|---|---|
| PR | 7 (17%) | 20.5 (13.3-28.6) | 26.8 (18.7-95.0) |
| MR | 3 (7%) | 23.7 (18.1-29.4) | 53.5 (28.1-79.0) |
| NC | 9 (21%) | 31.3 (16.0-61.4) | 65.2 (22.4-82.0) |
| PD | 23 (55%) | - | 10.8 ( 3.0-43.1) |

No. of patients evaluable for response = 42. PR, partial response; MR, minimal response; NC, stabilisation of disease; PD, progressive disease.

Table III depicts objective responses according to the type of primary cancer. No response or stabilisation were obtained in pancreatic carcinoma ($n$ = 5), gall bladder carcinoma ($n$ = 3) and epatocarcinoma ($n$ = 3), while four PR (33%) were obtained in gastric carcinoma ($n$ = 12). Out of 18 patients with colorectal carcinoma three (17%) achieved PR, two patients (11%) MR, six NC (33%) and seven patients (39%) progressed. Out of 10 patients previously treated with 5-FU containing regimens, three patients (30%) achieved an objective response (PR), three (30%) did not progress (NC) and four (40%) showed no response.

The impact of tegafur therapy on survival is shown in Table II and Figure 1. Patients who had PR (median survival 26.8 weeks) did not survive longer than patients who had a stabilisation of their disease (median 53.5 weeks). The difference in median survival between patients who responded (PR + MR) and those who progressed (median survival 10.8 weeks) is statistically significant ($P<0.001$).

Out of 50 enrolled patients, 44 (88%) were evaluable for toxicity. Toxic effects of oral TG according to WHO criteria are shown in Table IV. During a total of 161 complete cycles administered, the most frequent side-effects were: grade 1-3 nausea/vomiting in 48% of patients (only one case of grade 3), and grade 1-2 neurological toxicity in 25% of cases, mainly in the form of dizziness, headache, insomnia and lethargy. Neurological toxicity was generally mild and in no case was it dose-limiting. No renal and cardiac toxicities were seen.

**Figure 1** Survival of patients treated with oral TG according to objective response. Patients who had a partial response (median survival 26.8 weeks) did not survive longer than patients who had a stabilisation of disease (median survival 53.3 weeks). The difference in median survival between patients who responded and those who progressed (median survival 10.8 weeks) is statistically significant ($P<0.001$).

**Table III** Response according to primary neoplasm

| *Type* | *No. of patients* | *Type of response* | | | |
|---|---|---|---|---|---|
| | | *PR* | *MR* | *NC* | *PD* |
| Stomach | 12 | 4 (33%) | 1 (8%) | 3 (25%) | 4 (33%) |
| Pancreas | 5 | 0 | 0 | 0 | 5 (100%) |
| Liver | 3 | 0 | 0 | 0 | 3 (100%) |
| Gall bladder | 4 | 0 | 0 | 0 | 4 (100%) |
| Colorectal | 18 | 3 (17%) | 2 (11%) | 6 (33%) | 7 (39%) |
| Total | 42 | 7 (17%) | 3 (7%) | 9 (21%) | 23 (55%) |

**Table IV** Toxicity from oral tegafur

| | |
|---|---|
| No. evaluable patients | 44 (100%) |
| No. of patients without any toxicity | 13 (29%) |
| Gastrointestinal | |
| nausea/vomiting | 21 (48%) |
| grade 1–2 | 16 (36%) |
| grade 3 | 5 (11%) |
| diarrhoea | 4 (9%) |
| grade 1–2 | 3 (7%) |
| grade 3 | 1 (2%) |
| Haematological | |
| WBC | 7 (16%) |
| grade 1–2 | 7 (16%) |
| grade 3 | 0 |
| PLT | 0 |
| Hb | 2 (4%) |
| grade 1–2 | 2 (4%) |
| Neurological | 11 (27%) |
| grade 1 | 4 (12%) |
| grade 2 | 7 (17%) |
| Cutaneous pigmentation | 2 (6%) |
| Alopecia | none |
| Cadiac | none |
| Renal | none |
| Therapy-related death | none |

### Discussion

Tegafur (TG) is an anti-metabolite closely related to 5-fluorouracil (5-FU) and 5-fluorodeoxyuridine (5-FUDR). Clinical studies have demonstrated that TG exert an antineoplastic activity comparable to that of 5-FU against several tumours, including gastrointestinal and breast carcinomas (Buroker *et al.*, 1977; Friedman & Ignoffo, 1980; Schutt *et al.*, 1983). Full dose infusion of TG is often associated with severe gastrointestinal and neurological toxicity, which makes the drug unsuitable for repeated intravenous administration. TG, however, is reliably absorbed by the gastrointestinal tract and low dose oral therapy for 14–21 consecutive days minimises the toxic effects seen after infusion (Friedman & Ignoffo, 1980; Hunter & Browder, 1980; Bedikian *et al.*, 1983).

We treated 50 consecutive patients affected by advanced and/or metastatic gastrointestinal tract cancer (GTC) with oral TG 1,000 $mg\,m^{-2}$ on days 1–14. This regimen was repeated every 28 days or until toxicity recovered. The treatment was generally well tolerated. Mild neurological and gastrointestinal toxicities were seen respectively in 25% and 48% of patients. Seventeen per cent of 42 evaluable patients achieved a partial response, 7% had a minor response, 21% showed a stabilisation of disease and 55% of patients progressed. This overall response rate confirms our preliminary results (Palmeri *et al.*, 1986) and is within the range of activity reported by other authors for oral TG (Browder *et al.*, 1979; Stroehlein *et al.*, 1981; Ansfeld *et al.*, 1983; Brenner *et al.*, 1989). A 17% overall response rate was achieved in 18 patients with colorectal cancer, and a 33% response rate was obtained in patients with gastric carcinoma. No objective response was seen in pancreatic, gall bladder and liver carcinomas. Although Piccinini *et al.* (1986) reported a 64% partial response rate in liver carcinoma, our results suggest that oral TG is ineffective against carcinomas of the hepatobiliary tract and the pancreas, while it is effective in gastric and colorectal carcinomas. Since all PRs were obtained in patients with secondary hepatic neoplastic lesions, oral TG seems to be particularly effective against hepatic metastasis.

Three (30%) out of 10 patients previously treated with 5-FU-containing regimens also responded to oral TG, suggesting a possible lack of cross-resistance of TG and 5-FU. This observation is consistent with the results of Ansfield *et al.* (1980), reporting an objective response in 50% of patients pretreated with 5-FU and progressed thereafter. However, these data are not confirmed by experimental studies which demonstrated cross-resistance of TG and 5-FU in mice bearing L1210 lymphocytic leukaemia (Garibjarian *et al.*, 1976). Initial pharmacological studies showed that TG is a pro-drug of 5-FU for it is slowly metabolised in the liver by microsomal enzymes to 5-FU (Belitsky *et al.*, 1981). 5-FU is in turn slowly released into the systemic circulation where it reaches detectable levels for a prolonged period of time (Garibjanian *et al.*, 1976; Benvenuto *et al.*, 1978; Schilcher *et al.*, 1983). However, other reports have demonstrated that 5-FU plasma concentrations after TG administration are almost undetectable and considerably below those observed after an equivalent intravenous dose of 5-FU (Au & Sadee, 1979; Au *et al.*, 1979; Hornbeck *et al.*, 1981). It seems likely that TG is intracellularly converted to 5-FU which is further metabolised before redistribution. As reported by Au *et al.* (1979), alternative routes of intracellular activation of TG to 5-FU with the production of active metabolites cannot be excluded at present. Moreover intracellularly formed 5-FU may be further metabolised without being redistributed through the circulation (Au *et al.*, 1979).

Statistical analysis failed to show any significant difference in survival between patients who enjoyed PR and those who had a stabilisation. On the other hand, patients who had PR survived longer than those who progressed ($P < 0.001$).

In conclusion our data suggest that oral therapy with TG is an active treatment for advanced and/or metastatic gastric and colorectal carcinomas with mild gastrointestinal and neurological toxicity, but without a striking positive impact on survival. No activity was seen in cancers arising from hepatobiliary system and pancreas. Although data concerning the lack of cross-resistance between 5-FU and TG are not conclusive, we feel that oral TG, alone or in combination with other drugs, may represent a useful drug in the palliative treatment of gastric and colorectal carcinomas. The employment of TG in cases pretreated with 5-FU is still a matter of debate.

### References

AISFIELD, F.J., KALLAS, G. & SINGSON, J. (1983). Phase I–II studies of oral tegafur (ftorafur), *J. Clin. Oncol.*, **1**, 107.

AISFIELD, F.J., KALLAS, G., SINGSON, J. & UY, B. (1979). Phase I–II clinical studies with i.v. and oral ftorafur, a preliminary report. *Proc. Am. Soc. Clin. Oncol.*, **20**, 349.

AISFIELD, F.J., KALLAS, G., SINGSON, J. & UY, B. (1980). Further phase I–II studies with oral tegafur (T) (ftorafur). *Proc. Am. Soc. Clin. Oncol.*, **21**, 347.

AU, J.L. & SADEE, W. (1979). 5-fluorouracil concentrations in human plasma following R,S-1(tetrahydro-2-furanyl)-5-fluorouracil (ftorafur) administration. *Cancer Res.*, **39**, 4289.

AU, J.L. & SADEE, W. (1980). Activation of ftorafur (R,S-l-(tetrahydro-2-furanyl)-5-fluorouracil) to 5-fluorouracil and – butyrolactone. *Cancer Res.*, **40**, 2814.

AU, J.L., WU, A.T., FRIEDMAN, M.A. & SADEE, W. (1979). Pharmacokinetics and metabolism of ftorafur in man. *Cancer Treat. Rep.*, **63**, 343.

BEDIKIAN, A.Y., BODEY, G.P., VALDIVIESO, M. & BURGESS, MA. (1983). Phase I evaluation of oral tegafur. *Cancer Treat. Rep.*, **67**, 81.

BENVENUTO, J.A., LU, K., HALL, S.W., BENJAMIN, R.S. & TI LI LOO (1978). Disposition and metabolism of l-(tetrahydro-2-furanyl)-5-fluorouracil (ftorafur) in humans. *Cancer Res.*, **38**, 3867.

BELITSKY, G.A., BUKHMAN, V.M. & KONOPLEVA, I.A. (1981). Changes in toxic and antitumor properties of ftorafur by induction or inhibition of the microsomal enzymes activity. *Cancer Chemother. Pharmacol.*, **6**, 183.

BLOKHINA, N.G., VOZNY, E.K. & GARIN, A.M. (1972). Results of treatment of malignant tumors with ftorafur. *Cancer*, **30**, 390.

BRENNER, J., AMSTERDAM, E., CRISPIN, M., KAUFFMAN, M. & WEISBERG, D. (1989). Pilot study of one year continuous oral ftorafur for colon, rectum, and gastric cancers. *Proc. Am. Soc. Clin. Oncol.*, **5**, 127.

BUROKER, T., MILLER, A., BAKER, L., MCKENZIE, M., SAMSON, M. & VAITKEVICIOUS, V.K. (1977). Phase II clinical trial of ftorafur in 5-fluorouracil refractory colo-rectal cancer. *Cancer Treat. Rep.*, **61**, 1579.

BYFIELD, J.E., HORNBECK, C.L., FRANKEL, S.S., SHARP, T.R. & GRIFFITHS, J.C. (1985). Relevance of the pharmacology of oral ftorafur to its use as a 5-FU pro-drug. *Cancer Treat. Rep.*, **69**, 645.

CARTER, S.K. (1976). Large bowel cancer: the current status of treatment. *J. Natl Cancer Inst.*, **56**, 3.

CARTER, S.K. & COMIS, R.L. (1975). Adenocarcinoma of the pancreas, prognostic variables, and criteria of response. In *Prognostic Factors and Criteria of Response*, Staquet, M.J. (ed.) p. 237. Raven Press: New York.

CARTER, S.K. & SLAVIK, M. (1976). Investigation drugs under study by the United States National Cancer Institute. *Cancer Treat. Rev.*, **3**, 43.

COMIS, R.L. & CARTER, S.K. (1974). Integration of chemotherapy into combined modality treatment of solid tumors. III. Gastric cancer. *Cancer Treat. Rev.*, **1**, 221.

DIASIO, R.B., HUNTER, H.L., LA BUDDE, J.A. & MAYOL, R.F. (1979). Pharmacologic study of oral ftorafur: potential for improved oral delivery of 5-fluorouracil. *Proc. Am. Soc. Clin. Oncol.*, **20**, 401.

DINDOGRU, A., VAITKEVICIUS, V.J., YOUNG, J.D., HORWITZ, J.P. & BAKER, L.H. (1980). Pharmacological studies and phase I evaluation of oral ftorafur (FTF). *Proc. Am. Soc. Clin. Oncol.*, **21**, 167.

FRIEDMAN, M.A. & IGNOFFO, R.I. (1980). A review of the United States clinical experience of the fluoropyrimidine, ftorafur (NSC-148958). *Cancer Treat. Rev.*, **7**, 205.

GARIBJANIAN, T.B., JOHNSON, R.K., KLINE, I. & 4 others (1976). Comparison of 5-fluorouracil and ftorafur. II. Therapeutic response and development of resistance in murine tumors. *Cancer Treat. Rep.*, **60**, 1376.

HORNBECK, C.L., GRIFFITHS, J.C., FLOYD, R.A., GINTHER, N.C., BYFIELD, J.E. & SHARP, T.R. (1981). Serum concentration of 5-FU, ftorafur, and a major serum metabolite following ftorafur chemotherapy. *Cancer Treat. Rep.*, **65**, 69.

HUNTER, H.L. & BROWDER, H.P. (1980). Minimization of side effects with low daily dose, extended course tegafur (ftorafur). *Proc. Am. Soc. Clin. Oncol.*, **21**, 349.

JOHNSON, R.K., GARIBAJANIAN, B.T., HOUCHENZ, D.P. & 4 others (1976). Comparison of 5-fluorouracil and ftorafur. I. Quantitative and qualitative differences in toxicity to mice. *Cancer Treat. Rep.*, **60**, 1335.

LAI-SIM AU, J. & SADEE, W. (1981). Stereoselective metabolism of ftorafur (R,S-1-(tetrahydro-2-furanyl)-5-fluorouracil). *Cancer Chemother. Pharmacol.*, **7**, 55.

MORGAN, L.R., BROWDER, H. & CARTER, R.D. (1979). Oral ftorafur: a feasibility study. *Proc. Am. Soc. Clin. Oncol.*, **20**, 397.

PALMERI, S., GEBBIA, N., D'ALESSANDRO, N. & 4 others (1986). Ftorafur (FT) in gastrointestinal tract (GIT) cancer treatment: a phase II study. *Cancer Chemother. Pharmacol.*, **23**, suppl., 217.

PICCININI, L., CORRADINI, R., VANDELLI, C., LUPPI, G. & DIMARCO, G. (1986). Tegafur chemotherapy for the treatment of gut and liver cancer. *Int. J. Clin. Pharmacol. Res.*, **6**, 255.

SCHILCHER, R.B., YOUNG, J.D., LEICHMAN, L.P., SMITH, L.B., EVANS, L.J. & BAKER, L.H. (1983). Clinical and pharmacokinetic evaluation of tegafur in patients with or without metastatic liver disease. Proc. 13th International Congress of Chemotherapy, Wien, 28 August to 2 September. p. 22.

SCHUTT, A.J., HAHN, R.H., MOERTEL, C.G., O'CONNELL, M.J., RUBIN, J. & CREAGAN, E.T. (1983). Phase II study of ftorafur in previously untreated and treated patients with advanced colorectal cancer. *Cancer Treat. Rep.*, **67**, 505.

STROEHLEIN, J.R., BEDIKIAN, A.Y., KARLIN, D.Y. & 4 others (1981). A randomized study of oral ftorafur vs i.v. 5-fluorouracil in advanced colorectal cancer. *Proc. Am. Soc. Clin. Oncol.*, **22**, 451.

VAN PUTTEN, L.M., LELIEVELD, P., PANTAROTTO, C., SALMONA, M. & SPREAFICO, F. (1979). Ftorafur: a self limiting source of 5-fluorouracil? *Cancer Chemother. Pharmacol.*, **3**, 61.